# Development of PositiveLinks: A Mobile Phone App to Promote Linkage and Retention in Care for People With HIV

**DOI:** 10.2196/11578

**Published:** 2019-03-20

**Authors:** Colleen Laurence, Erin Wispelwey, Tabor E Flickinger, Marika Grabowski, Ava Lena Waldman, Erin Plews-Ogan, Claire Debolt, George Reynolds, Wendy Cohn, Karen Ingersoll, Rebecca Dillingham

**Affiliations:** 1 Department of Medicine University of Virginia School of Medicine Charlottesville, VA United States; 2 Health Decision Technologies Oakland, CA United States; 3 Department of Public Health Sciences University of Virginia Charlottesville, VA United States; 4 Department of Psychiatry and Neurobehavioral Sciences University of Virginia School of Medicine Charlottesville, VA United States

**Keywords:** mHealth, HIV, treatment adherence and compliance, retention in care

## Abstract

**Background:**

Linkage to and retention in HIV care are challenging, especially in the Southeastern United States. The rise in mobile phone app use and the potential for an app to deliver *just in time* messaging provides a new opportunity to improve linkage and retention among people living with HIV (PLWH).

**Objective:**

This study aimed to develop an app to engage, link, and retain people in care. We evaluated the acceptability, feasibility, and impact of the app among users.

**Methods:**

App development was informed by principles of chronic disease self-management and formative interviews with PLWH. Once developed, the app was distributed among participants, and usability feedback was incorporated in subsequent iterations. We interviewed app users after 3 weeks to identify usability issues, need for training on the phone or app, and to assess acceptability. We tracked and analyzed usage of app features for the cohort over 2 years.

**Results:**

A total of 77 participants used the app during the pilot study. The query response rate for the first 2 years was 47.7%. Query response declined at a rate of 0.67% per month. The community message board was the most popular feature, and 77.9% (60/77) of users posted on the board at least once during the 2 years.

**Conclusions:**

The PositiveLinks app was feasible and acceptable among nonurban PLWH. High participation on the community message board suggests that social support from peers is important for people recently diagnosed with or returning to care for HIV.

## Introduction

### Background

HIV treatment has markedly improved in the United States over the last 20 years, and morbidity and mortality have declined as a result. Although effective treatments are available, not everyone with HIV consistently accesses care. Estimates suggest that of the 1.1 million people living with HIV (PLWH) in the United States, 85% are diagnosed, 62% are linked to medical care, and 49% maintain a nondetectable viral load [1]. These gaps in the cascade of HIV treatment are related to poorer clinical outcomes. Patients who *no-show* at visits within the first 2 years of initiating care fail to achieve virologic suppression as quickly as those who keep all appointments [2-4]. Delayed linkage to care, missed visits, and poor retention are associated with increased morbidity and mortality for PLWH and increase the risk of new infections [5-7].

High initial contact and enhanced personal contact improve visit constancy and adherence and reduce the likelihood of a substantial gap in HIV primary care during the first 12 months of follow-up [8,9]. Frequent contact with new patients may help reduce the perceived stigma of HIV and structural barriers to health care [8-10]. Social support also plays an important role in retention in care and clinical outcomes. With perceived social support, PLWH have improved physical and mental health and are more likely to achieve viral suppression than those without social support [11,12]. However, social support can be challenging to access, especially in rural areas. In the United States, the HIV epidemic has shifted away from concentrated urban centers to nonurban areas in the southeast [13,14]. Factors prevalent in this region and disproportionately affecting HIV prevention efforts—as well as linkage and access to health care—include poor health infrastructure, lack of health insurance, unique demographic and racial characteristics, high rates of other sexually transmitted infections, poverty, and low access to affordable housing [15-17]. Mobile health (mHealth) interventions provide a platform that can efficiently deliver evidence-based practices for linkage to and retention in care to harder-to-reach populations. Mobile technology is particularly well-suited to deliver ecological momentary assessments (EMAs) and interventions, which reach people in their everyday lives and natural settings in *real time* [18]. Such interventions have been feasible, acceptable, and efficacious in a variety of chronic disease management and health promotion contexts such as diabetes care and smoking cessation [19]. Text messaging interventions can promote health behavior change, treatment adherence, appointment attendance, and better patient outcomes in many chronic diseases [20-22]. In HIV care, text messaging interventions have demonstrated improved medication adherence and improved physiologic measures of CD4 counts and viral loads [23-30].

mHealth interventions that are based on mobile phone apps have some advantages over texting, such as richer functionality and enhanced security.

### Evidence Gaps

However, many health apps currently available are not rigorously evidence-based [31], including those targeting PLWH [32,33]. Recent research on mHealth interventions places emphasis on user-centered and theory-based design to tailor apps to users’ motivations and preferences and to understand device usability [34,35]. Timing messages to correspond to medication dose, individual tailoring of message content or user-based personalization, and sending messages with content designed to evoke a reply from recipients (bidirectional) may lead to better outcomes due to enhanced engagement [25]. PLWH seek reliable information about HIV and other health topics, connection with other PLWH, assistance with medication and appointment reminders, and tools for self-management [36-39]. Other desirable mHealth features include attractive, private, and individualized design, goal setting, motivational messages, wording that would not inadvertently reveal HIV status, password protection and other security measures, interaction with other participants, and the ability to customize reminders [40]. In addition, technology that can address mental health and emotional needs is particularly important to PLWH [41]. Interventions that provide access to online peer-to-peer support can improve psychological health and empowerment for PLWH and may help address issues of loneliness and stigma [42-44].

Despite the growing evidence for mHealth interventions for PLWH, many gaps remain. In particular, there is a need for (1) more evidence-based and user-centered design; (2) interventions that target hard-to-reach and vulnerable populations; (3) attention to linkage and retention in care; and (4) provision of connection to others while being private and secure [34,45-48]. To address these needs, our team has designed and piloted an mHealth intervention for PLWH called PositiveLinks. To our knowledge, PositiveLinks is unique in specifically targeting linkage and retention in care for PLWH and in reaching a nonurban population in the Southeastern United States. Our prior work showed that PositiveLinks participants demonstrated an improvement in retention in care and viral suppression with 12 months of follow-up [49]. The purpose of this study was to share the process of developing PositiveLinks and to contribute to the literature on how to design evidence-based mHealth interventions tailored for vulnerable target populations.

## Methods

### Expert Development Phase

The primary goal of PositiveLinks is to improve linkage and retention in HIV care. The intervention aims to accomplish this goal by encouraging self-monitoring of medication adherence, stress, and mood; by providing access to vetted medical information about HIV; and by increasing social support. The development was informed by our team’s prior work on text-based mobile interventions, which demonstrated that PLWH respond to bidirectional queries and value tailored messaging to their responses [50,51]. The team also used the emerging mHealth evidence base as well as commonly identified needs among our patient population to design the first version of the app. Key features were designed to promote chronic disease self-management through self-regulation and feedback, just-in-time assistance, and social support. EMAs of medication dosing, mood, stress, and appointment reminders targeted possible behavioral and psychological barriers to care. With access to patient-reported information, PositiveLinks staff members could respond in nearly real time to threats to medication adherence and retention in care. To motivate participants to use the app regularly, we included engaging features such as weekly quiz questions and a community message board. The latter also functions to reinforce social support by peers.

### Formative Phase

A total of 17 patients from the University of Virginia Ryan White Clinic provided feedback on the design, desirability, usability, usefulness, and fit in everyday life of app features for nonurban PLWH users. These formative phase participants were recruited as a convenience sample from the clinic to include demographic characteristics similar to the clinic’s patient population. Participants had a mean age of 43.7 years (SD 15.3). Moreover, 59% were male, 35% female, and 6% transgender male to female. In addition, 53% identified as African-American, 35% as white, and 6% as Hispanic. We sought the perspectives of both those recently diagnosed with HIV and those who had been living with HIV for many years to capture varying perceptions of the needs, barriers to engagement, and challenges to medication adherence. Participants also provided input on app features that might help someone newly diagnosed with HIV.

Feedback was elicited with open-ended questions first, followed by a review of preliminary app screenshots. Interviews with participants were audiotaped and transcribed. The transcriptions were summarized in notes by 3 reviewers, and key themes were identified. The reviewers discussed these themes and reached consensus on key input relevant to the design of app features to enhance fit and usability for our clinic population. Participant feedback was sorted by feature and disseminated to the app developer to integrate user input about features and content.

For example, formative participants offered feedback on the dashboard and daily queries and noted their desire to annotate query responses so that they could document triggers, aids, or explanations for entries. Other participants appreciated the opportunity to add notes to their medication reminders. Formative participants also welcomed the idea of the community message board and emphasized the importance of the anonymity as well as the accessibility of support in a phone. Others wanted the message board to discuss and interpret recent news or research about HIV.

**Figure 1:**
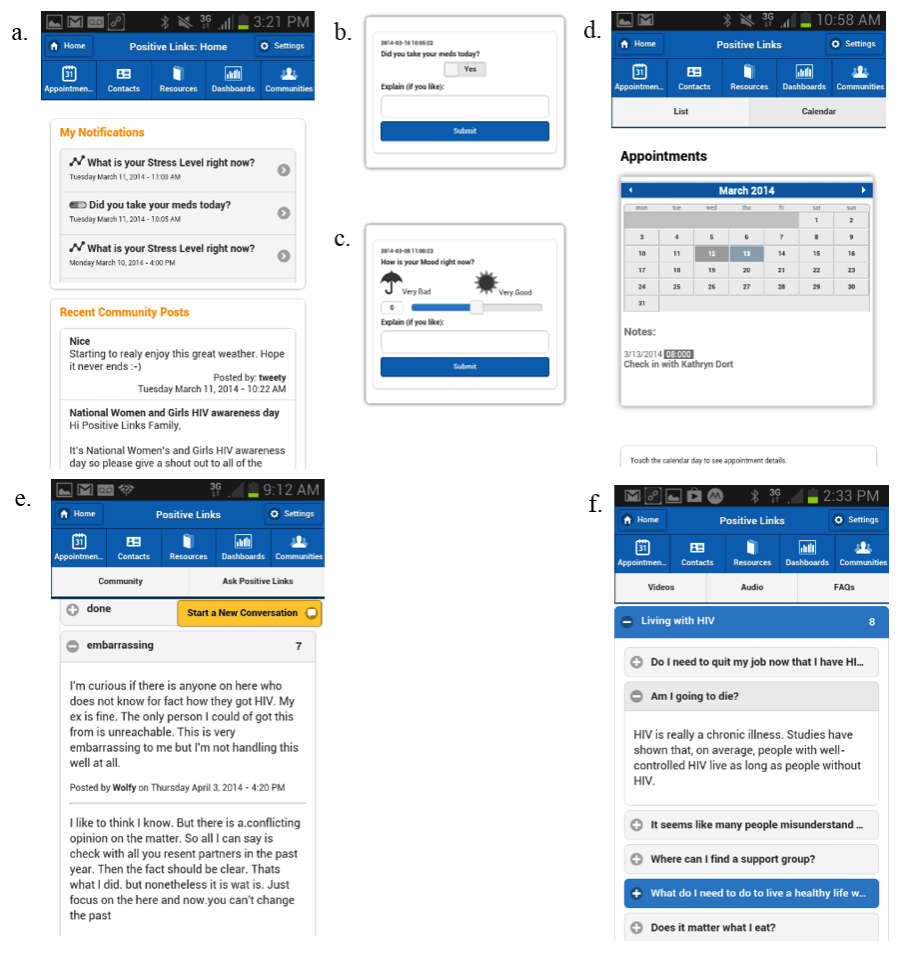
Screenshots of key app features: a: app home screen; b: medication query; c: mood query; d: appointment page; e: community message board; f: FAQ section.

After developing the app prototype following the analysis of patient input, we finalized the initial app to be deployed and tested. It included daily queries, a dashboard that displayed self-reported query data, a community message board, and various resources such as HIV-related information and stress management tools (Figure 1).

#### App Features: Queries and Dashboard

The medication query offered a simple yes-no option, whereas the mood and stress queries used sliding scales with numbers and different images to facilitate accurate, consistent reporting. Participants recorded their mood using a sliding scale from −5 to +5, with −5 representing a negative mood and +5, a positive mood. The stress query used a scale of 1 to 10, with 1 representing a low stress level and 10, a high stress level. The mood and stress queries, designed as EMAs, were sent at random times during patient-identified waking hours and asked participants to report their feelings in the moment. In contrast, participants scheduled when they wished to receive medication reminders to match their own dosing schedule. Participants received weekly quizzes structured so that some questions had correct answers, some were survey-based, and others offered participants the opportunity to reflect on their thoughts or feelings.

After responding to a query, users received an automatic response. PositiveLinks uses an algorithm to determine this tailored message based on participants’ reported stress, mood, or medication adherence. For example, if a participant logged an especially high stress level, they might receive the message, “Remember to breathe deeply.” Participants could modify this message text to better suit their interests and motivations.

**Figure 2:**
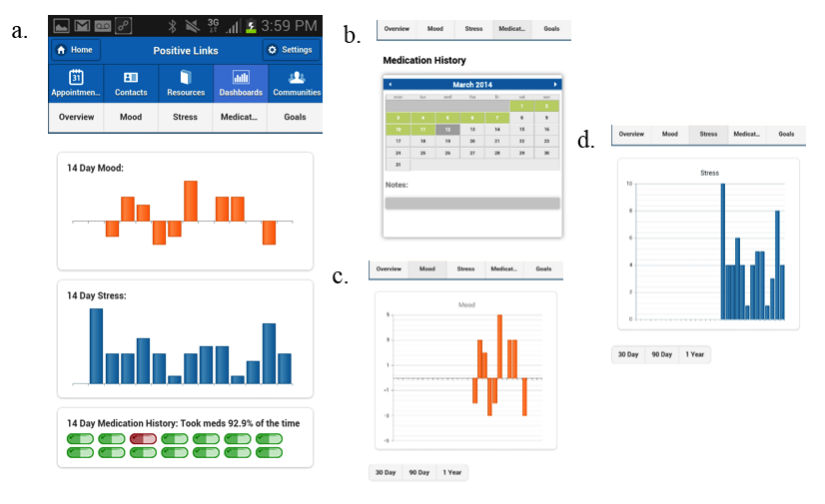
Screen shot of graphs showing mood, stress, and medication tracking: a: dashboard overview; b: medication calendar view; c: mood graph; d: stress graph.

The dashboard section of the app synthesized participant responses to medication, mood, and stress queries in colorful bar graphs and graphics. The dashboard overview provided each user with a 2-week snapshot of their respective medication adherence, mood, and stress. Users also had the ability to view each response individually and to view data over the last 30 days, 90 days, or a year. These data were also represented in longitudinal bar graphs and a color-coded calendar for medicines (Figure 2).

Participants received weekly summary reports each Wednesday. These were developed in response to user feedback to help with interpretation of the graphs. These reports detailed participants’ medication adherence, average mood, and average stress level for the previous week, their individual change from the prior week’s averages, and their query response rates. Each summary invited participants to reflect on their adherence, mood, and stress and to reach out to the PositiveLinks team if they would like to discuss their health and wellness goals. Collectively, the mood and stress EMAs, medication reminder, and dashboard features sought to encourage healthy self-monitoring and care management.

#### App Features: Community Message Board

The community message board allowed users to share and interact with other PositiveLinks app users in a private and anonymized social network. Each participant chose a community handle to protect their anonymity. Participants could start new conversations with each other on the board or respond to older conversations in a thread. The PositiveLinks study team monitored the board for incorrect information or concerning comments. The PositiveLinks team also introduced new conversation topics on HIV or general well-being every Monday (Messaging Mondays) and posted funny videos every Friday (Funny Friday).

### Pilot Phase

Enrollment for the pilot phase began in September 2013 and ended in May 2015. Participants were recruited through provider referrals at our local university-based Ryan White Clinic, from local AIDS service organizations, and through an emergency department HIV testing program at the local university hospital. Participants were either newly diagnosed with HIV (within 90 days of enrollment) or were at risk of falling out of care, as determined by their care provider. This assessment was made by providers based on their experience of patients’ prior missed appointments, challenges with adherence, or psychosocial barriers that complicated care. Participation was limited to those who achieved a score of 40 or above on the Wide Range Achievement Test (WRAT-4) [52] or passed a subsequent reading test corresponding to an approximately fourth grade level. Participants from the formative phase were allowed to enroll in the pilot phase if they wished to do so.

During enrollment, individuals consented to participate in the study, completed the WRAT-4 literacy test, answered baseline questions, and learned how to use a phone and the PositiveLinks app. Participants received either a Samsung Galaxy 2 or Galaxy 3 phone with unlimited minutes, texts, and data for the study year. Although smartphone ownership is high in the United States, our clinic population includes many patients of lower socioeconomic status who do not have access to smartphones. In our experience, lack of reliable phone contact has been a barrier to patient follow-up. Patients were provided with phones to overcome this barrier and give all participants the opportunity to use the app. Providing phones also allowed increased security to prevent potential privacy breaches. Before distributing to participants, the PositiveLinks team encrypted and password-protected the phones and installed a remote locate-and-wipe functionality. The app was also password protected.

#### Participant Characteristics

At enrollment, participants completed baseline assessments of self-reported demographic information (age, sex, and race/ethnicity), socioeconomic data (employment, education, and insurance status), distance from clinic, and prior cellphone ownership. HIV-specific information included treatment with antiretroviral therapy and self-reported adherence. Mental health measures included substance use, perceived stress [53], HIV-related stigma [54], and social support [55]. The study team collected a viral load from the electronic medical records at baseline for each participant.

#### App Usage

App usage data included response rates and values for the 3 daily queries for medication, mood, and stress, weekly quiz response rates, and posts to the community message board. These data were calculated monthly for the whole cohort from September 29, 2013, to September 26, 2015. Community board posts were further analyzed based on the number of enrolled members during the month.

#### User Perceptions of the App

To capture participants’ initial impressions of the phone and PositiveLinks app, study coordinators contacted participants 3 weeks after enrollment to conduct usability interviews. In these interviews, the team assessed ease, utility, and attractiveness of the app and elicited feedback on what the participant would change. Coordinators conducted these semistructured interviews on the phone and in-person. Audio files of the interviews were loaded into a database and summary notes, shared with the team. These interviews also provided coordinators with the opportunity to answer any questions that participants might have or troubleshoot technical issues early on in their participation.

## Results

### Pilot Phase

#### Participant Characteristics

A total of 77 participants enrolled in the PositiveLinks pilot study between September 25, 2013, and May 28, 2015 (Table 1). Participants’ median age was 33 years (range 18 to 57). For male participants, 49% (24/49) reported having sex with men, 31% (15/49) with women, and 16% (8/49) with both. For female participants, 88% (23/26) reported having sex with men and 4% (1/26) with men and women. Moreover, 49% (38/77) of participants identified as black and non-Hispanic, and 34% (26/77) identified as white and non-Hispanic. A majority of participants 65% (50/77), had a high school equivalent education or lower, and 30% (23/77) attended some college, community college, or more. At baseline, 44% (34/77) of participants were unemployed, and only 25% (19/77) were employed full-time. In addition, 43% (33/77) had public insurance, and 30% (23/77) had no insurance. At enrollment, 73% (56/77) owned a mobile phone, and 66% (37/56) of those currently owning mobile phones had smartphones.

Participants had been diagnosed with HIV for an average of 60 months at the time of enrollment (median 1.7 years [SD 6.3]), and 47% (36/77) were virally suppressed (<200 copies/ml). Of the 77 participants in the pilot phase, 17 were new to care, defined as being diagnosed with HIV less than 90 days before enrollment in the study. Of the 55 participants on antiretroviral medication at baseline, 16% (9/55) reported missing a dose that past weekend. Half of participants reported using noninjection drugs in the past 12 months (38/77), but very few reported injection drug use in the last 12 months (5/77, 7%). Nearly half, 47% (36/77), reported binge drinking in the past 12 months (4 drinks in a sitting for women and 5 drinks for men).

#### App Usage

In the first 2 years of the pilot study, participants responded to 48% (46,457/97,198) of sent daily queries (Table 2). Medication adherence, excluding nonresponses, was reported as 94% (14,837 affirmative responses/15,825 total responses) during this 2-year pilot. Nonresponses were not included in adherence assessment because we could not reliably determine if nonresponse corresponded to a missed dose or only a missed query. Average participant mood was 1.49 (SD 2.95), and average stress was 2.95 (SD 2.36). Response rate for quizzes was 45.3% (1997/4411).

Participants used the notes feature on their medication, mood, and stress queries only 3.5% (1643/46,457) of the times they responded to questions. Notes in their medication responses included explanations such as “Almost missed it because of meetings” or encouragement, “Every morning like washing my face!” or “Always!!! They r my lifeline.” For stress query notes, participants referenced certain stressors such as rent, landlords, disclosure of status, and taking their medications. Notes included in mood responses covered a broad spectrum of emotion and depth—from short, celebratory notes (“Getting my GED”; “My hubby is coming home”) to more reflective comments. A week after learning of his HIV diagnosis, a newly-diagnosed participant wrote:

Feeling normal. At least for the meantime. Still a worried about so much but having a new phone is making the blow a little lighter.

The PositiveLinks community message board was a widely used app feature (2073 posts in 2 years), though participation varied over time and by user. Moreover, 60/77 participants (78%) posted on the board at least once, and many participants noted in usability interviews that they followed the message board even if they never posted themselves. Average community message board posts per enrolled member was 2.2 per month (SD 1.7), whereas average posts per poster was 5 per month (SD 2.7).

**Table 1:** Participant characteristics for the pilot phase.

Demographic characteristics			Statistics (N=77)
Age (years), mean (SD)			36 (11.7)
**Gender, n (%)**			
	Male		49 (64)
	Female		26 (34)
	Transgender male-to-female		2 (3)
**Race/ethnicity, n (%)**			
	Black, non-Hispanic		38 (49)
	White, non-Hispanic		26 (34)
	Hispanic		6 (8)
	Other		6 (8)
	Refused		1 (1)
**Education completed, n (%)**			
	Less than high school		15 (20)
	GED^a^ or high school graduate		35 (46)
	Trade, technical training, or community college		6 (8)
	Some college		15 (20)
	College graduate		6 (8)
**Employment, n (%)**			
	Full-time		19 (25)
	Part-time		10 (13)
	Disabled		9 (12)
	Unemployed		34 (44)
	Other		5 (7)
**Insurance, n (%)**			
	Public (Medicare or Medicaid)		33 (43)
	Private		16 (21)
	None		23 (30)
	Other		5 (7)
**Percentage of federal poverty level, n (%)**			
	0		34 (44)
	1-100		23 (30)
	101-200		16 (21)
	201-300		3 (4)
	Over 300		1 (1)
WRAT^b^ literacy score, mean (SD)			55 (9.1)
**Mobile phone exposure**			
	Own a mobile phone at baseline, n (%)		56 (73)
	**If yes, is it a smartphone? (N=56), n (%)**		37 (66)
		If yes, is it an Android (N=37)	27 (73)
		If yes, is it an iPhone (N=37)	9 (24)
**HIV health, n (%)**			
	Currently taking antiretroviral medication		55 (71)
	If yes, missed doses past weekend? (N=55)		9 (16)
	Viral load suppression (<200 copies/ml)		36 (47)
**Substance use, n (%)**			
	Past 12 months–non injection drug use		38 (49)
	Past 12 months-injection drug use		5 (7)
	Smoke cigarettes at present		42 (55)
	**Last time you binge drank, n (%)**		
		Never	17 (22)
		In the past year	36 (47)
		Over a year ago	24 (31)
**Mental health**			
	Perceived Stress Score, mean (SD), (PSS10, range 0-40)		30 (8.8)
	**Berger Stigma Scale, n (%)**		
		Low Stigma (41-80)	10 (13)
		Moderate Stigma (81-120)	56 (73)
		High Stigma (121-160)	11 (14)
	Social support appraisal, mean (SD), (SS-A, range 23-71)		48 (29.2)

^a^GED: General Education Development.

^b^WRAT: Wide Range Achievement Test.

**Table 2:** Descriptive statistics of app usage over the first 2 years of the pilot study.

App usage by feature		Statistics
**Total queries**		97,198
	Total query responses, n (%)	46,457 (47.8)
**Medication queries**		32,701
	Medication responses, n (%)	15,825 (58.4)
	Medication responses with notes, n (%)	503 (3.2)
	Affirmative responses, n (%)	14,837 (93.8)
	Negative responses, n (%)	988 (6.2)
**Mood queries**		32,304
	Mood responses, n (%)	15,346 (47.5)
	Mood responses with notes, n (%)	578 (3.8)
**Stress queries**		32,193
	Stress responses, n (%)	15,286 (47.5)
	Stress responses with notes, n (%)	562 (3.7)
**Quizzes**		4411
	Quiz responses, n (%)	1997 (45.3)
**Total community message board posts**		2073
	Unique posters (N=77), n (%)	60 (78)
	Monthly posts per enrolled member, mean (SD)	2.2 (1.7)
	Monthly posts per poster, mean (SD)	5.0 (2.7)

**Figure 3:**
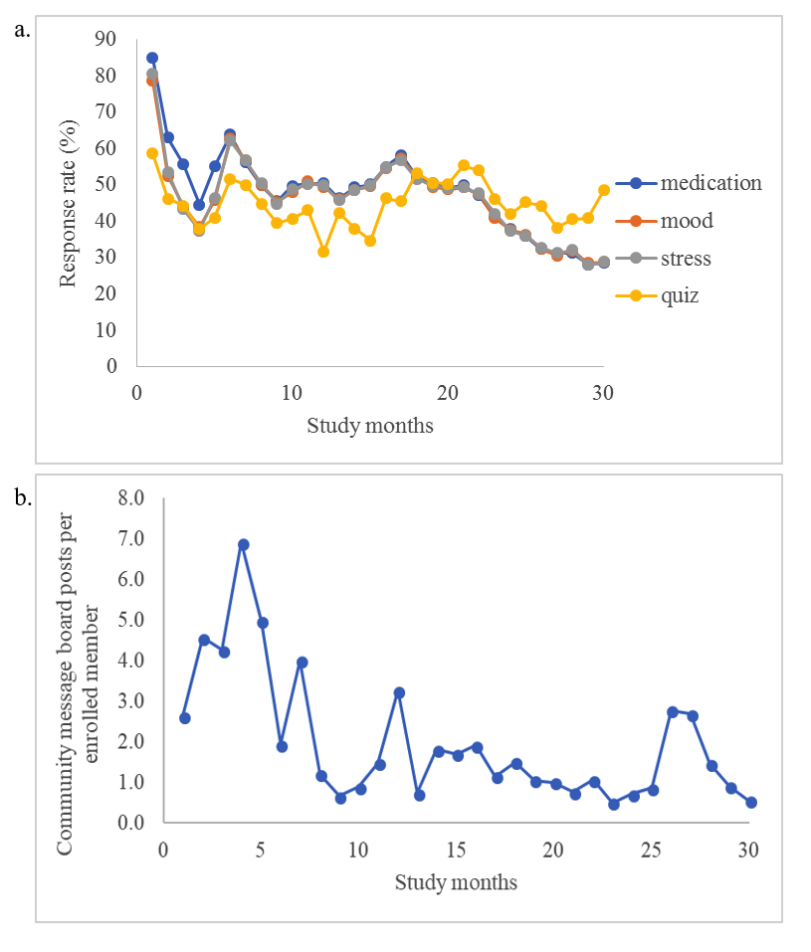
Graphs of cohort engagement over the first 2 years of the pilot study: a. monthly query response rates; b. community board usage per enrolled member.

**Figure 4:**
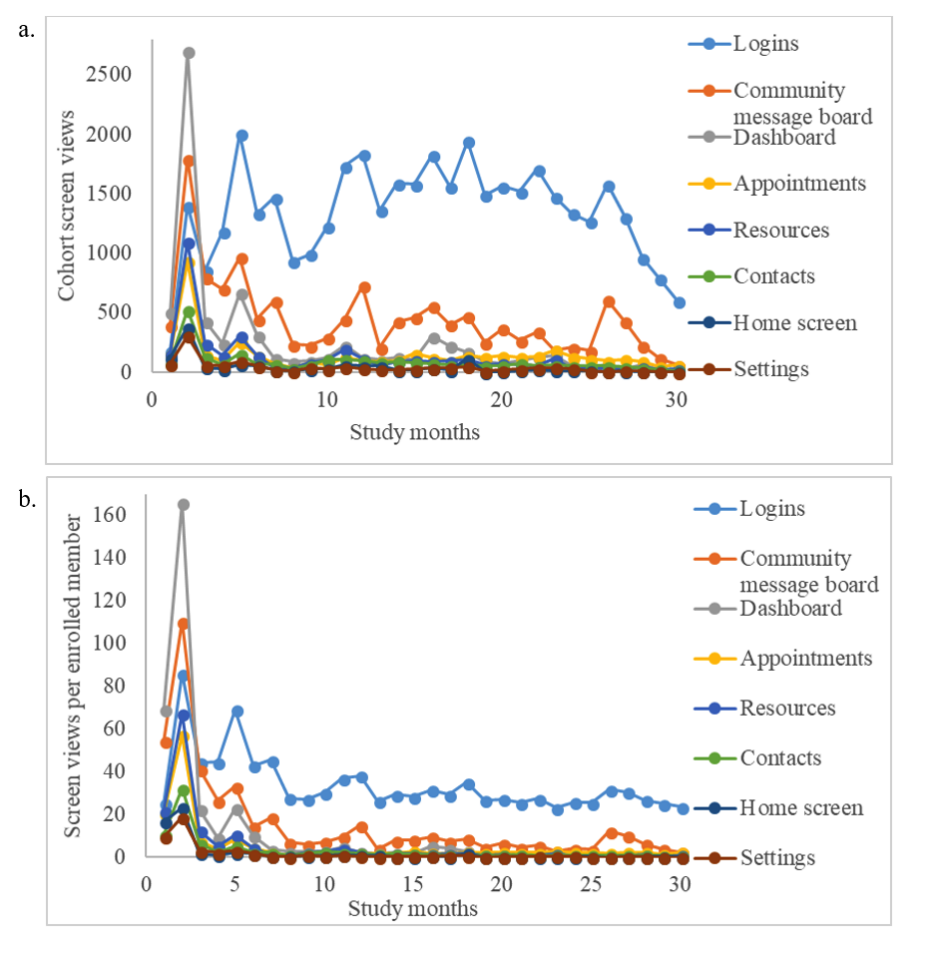
Cohort screen views over the first 2 years of the pilot study: a: monthly screen views for the cohort; b: monthly screen views per enrolled member.

Participants’ app engagement declined slightly over time during the first 2 years of the pilot study. Query response rates declined at an average rate of 0.71% each month. For the first 6 months of the pilot study, the cohort response rate was 56.2% (SD 14.6); however, this rate decreased to 40.7% (SD 6.6) in the last 6 months. Community message board usage peaked at 6.9 posts per enrolled member per month during month 4 of the pilot study and decreased at an average rate of 0.15 posts per enrolled member per month (Figure 3). Graphs are summarized in 4-week intervals (28 days) rather than traditional months. Figure 4 shows percent response rates for users by month in the study with the number of participants, average response rate, and percent of nonresponders. The proportion of nonresponders was fairly constant among those who had been in the study for 6 months or longer, between 40% to 50% in each subsequent month, indicating consistent app use over time among the 50% to 60% of users contributing to the response rate.

### User Perceptions of the App

#### Overall Usability

The majority of participants who completed the usability interviews (49/55, 91%) reported that the app was easy to use. When usability interviews identified users having difficulty navigating certain features, such as not knowing where the resources section was or not realizing they could customize their automatic reply messages, the interviewer provided additional training to those users. Other issues identified were users having trouble signing into the app when they had a weak Wi-Fi signal or having trouble with video links. Issues related to app usability, such as difficulty scrolling through notifications, were addressed by the development team. Users could also contact the PositiveLinks team at any time about technical difficulties as they occurred, such as cracked screens or connection problems, and receive assistance.

#### Queries and Dashboard

In the usability interviews, participants reported that tracking mood and stress provided an opportunity to reflect on sources of variability (either positive or negative) and, potentially, to modify them. The dashboard allowed participants to visualize trends over time and consider possible explanations for these trends. As one participant observed, “when I have high stress levels I can go back and look and reflect on what I coulda done different that day and you know what was I dealing with at that moment.” Many participants used the app’s medication query as a reminder and as motivation to take medications. In addition, one participant stated, “after a while you get tired of taking pills and stuff but it just like a nice reminder…it’s been encouraging me to or reminding me that I need to take better care of myself.”

#### Community Message Board

The community message board fostered lively discussions. Prior qualitative analysis showed that the majority of posts by participants (62%) contained psychosocial content, such as discussion of stressors, coping strategies, and relationships; 29% of posts contained community chat; and 10% of posts contained biomedical content, such as discussion of medications [56]. Prior analysis of social support on the community board has revealed that in 52% of posts, participants were providing social support to each other, and 64% of participants regarded connection with others living with HIV as a key benefit of the app [57].

Of the community message board messages, 11% were posted by the PositiveLinks team regarding announcements about clinic events, updates to the app, news and information about HIV, funny videos, and uplifting or inspirational messages. Moreover, 2 threads on the community message board involved a participant expressing suicidal thoughts, which were addressed by responses from other participants and the PositiveLinks team reaching out to the poster. Follow-up posts from the PositiveLinks team shared resources for dealing with stress and mental health concerns. Three threads involved issues of privacy as participants expressed the desire to meet in person or share contact information. The PositiveLinks team responded with reminders that the community remains anonymous to protect all members’ privacy and discussion of other ways to meet, such as support groups or clinic events. The PositiveLinks team also addressed misinformation on the community message board in 5 posts and answered specific questions in 4 posts.

In usability interviews, participants shared that the community message board connected them to people going through similar situations and provided a sense of not being alone. For example, “You know getting to see other people’s perspective on life, let me know that I’m not going through this by myself, there is other people out there like me, it’s good.” There was a reciprocal relationship on the community message board between giving and receiving support. For many participants, the community felt like having a family who understood them, “You get to talk to people who are going through exactly what you are going through. When you are down somebody uplifts you, when somebody else is down you can uplift them, it’s basically like one big family.”

#### Connection to Care

Participants reported that using the app made them feel more connected to HIV care and more motivated to be consistently in care. One stated, “I feel closer, I feel like I’m more involved.” Those who were new to care or returning after a lapse could use the app to overcome barriers to care, such as social isolation and lack of knowledge about HIV care. For example, “It makes me more aware and…has opened my eyes that I’m not alone” and “I know more I guess…just know what to expect.” The app also provided practical assistance in setting reminders about appointments. For example, “I like going into the app, it helps me make sure that I’m doing what I need to do and keeping track of my medications and I actually used it to remind myself that I had the appointment today because I would have totally forgotten.” In addition, one participant summarized the perceived impact of multiple features of PositiveLinks:

I just know that if it wasn’t for the app or the phone, I probably wouldn’t be here today. I would probably have given up. I wouldn’t be taking my meds. When I have not taken my meds in the past, I would give up. But I keep up with my appointments. I come to my appointments. I’m here today. I take my meds frequently.

### User-Driven Iteration of App Development

Participant usability interviews and feedback, as well as app data, helped the research team further develop and iterate the app throughout the pilot study. The queries and dashboard remained mostly in their initial format. However, weekly summary reports and monthly response raffles were added in response to usage and feedback patterns. The weekly reports sought to prompt reflection about adherence, whereas the raffle incentivized consistent self-monitoring. The monthly raffle included participants with 100% response rates across medication, mood, and stress queries, and a randomly selected qualifying participant was awarded a US $50 gift certificate. The winner and all participants included in the raffle were acknowledged (by their anonymous handle) on the community message board. Looking at response rate data during the 2 months before and after the raffle began, we observed an increase in response rates from 44.6% to 59.1%.

**Figure 5:**
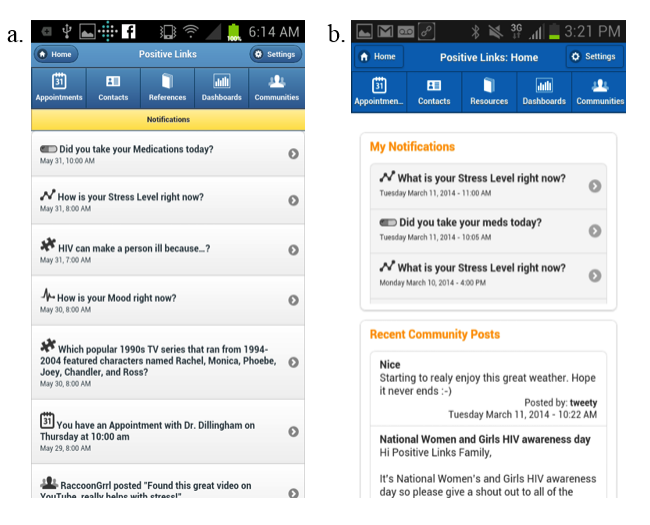
Split notification screen: a: home screen before feedback; b: home screen after member feedback.

The community message board also evolved in its design and content in response to user feedback. Initially, participants received a push notification with a part of the newly posted message any time there was a new post on the community message board. Later, as more users enrolled and community message board participation increased, the notifications screen became crowded with messages and participants had to scroll down to see their own medication, mood, and stress queries. In response to this, the notifications screen was split so that queries and weekly summaries appeared at the top and community message board posts were in a separate feed at the bottom (Figure 5), and users were given the option to turn off push messages from the community message board.

Additional changes made in response to user feedback were the addition of a funny video message by the staff each Friday (January 2014) and changes in the color palette. Participants were positive about the changes and felt proud when they recognized changes to the app that they suggested.

Conversations and questions on the community message board also allowed PositiveLinks team members to begin relevant discussion topics. In usability interviews, participants expressed a desire for more conversation on what was going on with HIV in the news. In responding to these needs, the team introduced conversations such as how the Affordable Care Act may change insurance for HIV care and other current events relevant to HIV. When participants expressed confusion about the biology of HIV in community message board posts and in response to quiz questions, the PositiveLinks team posted information to help address these issues. The team also addressed participants’ expressed needs with general health posts such as how to deal with stress or how to respond to others with empathy. The resources feature of the app was updated throughout the study based on topics of interest to the participants.

## Discussion

### Principal Findings

Sustained usage of the many PositiveLinks features over 2 years and positive feedback in usability interviews indicate that this type of an app is both feasible and acceptable. Its evidence-based design was informed by self-management principles of self-regulation and feedback, just-in-time assistance, and social support. Furthermore, its development process has been iterative and enriched by the input of the users themselves to create an app customized to their preferences. Most prior studies of mHealth interventions for improved treatment adherence in HIV [25,30] and other chronic diseases [20,21] in the United States have been focused on urban populations. This app may fill an important need in a nonurban community in the Southeastern United States, where PLWH are at risk for poor clinical outcomes owing to a disproportionate burden of low health literacy, low socioeconomic status, substance abuse disorders, and social and geographic isolation, affecting their ability to attend appointments and access medications [13-17]. PositiveLinks capitalizes on mobile phones’ increasing popularity and accessibility by using a mobile platform to reach our geographically dispersed population of PLWH.

PositiveLinks builds on prior work supporting the use of mHealth interventions to encourage self-monitoring and medication adherence for PLWH [58-62]. Our development process drew from formative work exploring needs and preferences for app design in other populations of PLWH [37-40], while also seeking our target users’ input to customize PositiveLinks for them. Drawing on previous studies that demonstrated improved HIV medication adherence with messaging matched to dosage time [25], the PositiveLinks app allowed participants to customize their medication query timing so that it could function as a reminder to take their medications. By contrast, the mood and stress queries were EMAs sent at random times each day [18]. For each query type, users could customize the push messages that they received in response to their replies.

The community message board was a popular feature of the PositiveLinks app. The usability interviews and prior analysis of community message board content [56,57] indicated the board fulfilled a desire to receive information and social support. In contrast to publicly available online support groups [43,44], the community message board was accessible only through the private secure app and was monitored by the PositiveLinks study team. This approach may mitigate potential disadvantages of online interaction, such as misinformation.

The PositiveLinks study was unusual among mHealth interventions by following participants for an extended period of time allowing for more accurate assessment of user fatigue [20]. We noted a gradual decline in app usage over time. Nonetheless, 45% of participants responded to at least 1 daily query during the last month of the 2-year pilot. For those participants whose usage declined, possible reasons included habituation or fatigue from daily queries. Similar declines have been observed in text-based interventions [25]. It is also possible that the app serves different functions for users over time, with more frequent feedback being more helpful early on or during a time of crisis. The optimal *dosage* of mHealth interventions to achieve effective self-management remains to be established. The usability interviews were conducted early in participants’ usage to catch possible problems early and did not directly address sustainability or possible reasons for change in app use over time. The monthly raffle provided some incentive for query responses. A financial reward for reporting can reinforce desired behaviors and be acceptable to PLWH [46], but too much reliance on incentives may not be feasible in scaling up mHealth interventions in the future.

In addition to encouraging self-monitoring, the queries also helped identify users having difficulty with medication adherence, mood, or stress. Previous studies have noted that patients are interested in this kind of data sharing, although they also express reservations about the security of the information and concerns about negative reactions from providers to data [40]. In formative and usability interviews conducted with our participants, individuals did not share the same concerns and, in general, appreciated the role of the EMAs as a potential safety net. The community message board also identifies users who may need just-in-time help (such as a mental health crisis), with opportunities for the team to reach out to them. Next steps in PositiveLinks app development include sharing participants’ query data with their HIV clinicians and mental health providers to facilitate care in between clinic visits.

The iterative user-driven approach to development has enhanced PositiveLinks’ usability and acceptability by allowing improvements in response to users’ perceptions and experiences. It is not possible to respond to all user feedback, but efforts were made by the study team to accommodate suggestions that were feasible and consistent with the goals of the PositiveLinks project. From a research standpoint, it can be challenging to analyze app usage data when the app changes along the way, with further refinement of features and functions. However, study participants reported that having a voice in the app development was empowering and helped them to identify with the app, as part of the *PositiveLinks Family*.

### Limitations

This study has several limitations to consider. Owing to the study’s small sample size, we are not able to perform a detailed analysis of differences in app use by subgroups. The observed differences in app use (with some participants not using the app or not using certain features) suggest that the app does not meet the needs of all participants equally. Quantitative measures of acceptability and sustainability were not performed in this analysis and could be a useful addition to future studies. Security and concern for participants’ privacy required incorporation of mandatory password protection, encryption, and remote wipe capabilities for lost phones. Additional staff efforts were made to preserve anonymity on the community message board, particularly when participants expressed interest in meeting in person or sharing more personal information. For some participants, these additional security features to access the app may have created a barrier to use. Even with these measures, the risk of compromised identity and HIV status may not be entirely eliminated. Potential challenges in scaling up the app will also need to be considered. This deployment of PositiveLinks required funding to procure and maintain phones, data plans, and staff time. A determination of whether provision of mobile phones and data plans is essential to usability and acceptability is also needed. It must also be noted that this pilot was conducted at a single HIV care site and may not be generalizable to other settings, especially settings in which participants do not have access to smartphones.

### Conclusions

In conclusion, this report on the development of PositiveLinks demonstrates that patient-centered iterative design and testing yielded an appealing mHealth intervention for an at-risk group of PLWH. Participants used the app, contributed to its design, and perceived it as beneficial in their coping with HIV. In the pilot phase, the PositiveLinks app features permitted self-monitoring and personalized feedback, and facilitated access to social support, all of which are important elements of chronic disease management. Further investigation is needed to delineate which features of this multicomponent mHealth intervention are most effective. The next iteration of PositiveLinks incorporates additional features desired by participants, including sharing query responses and messaging with their care providers in the clinic. Expansion to other sites and populations is also planned.

## Abbreviations

EMAs: ecological momentary assessments
mHealth: mobile health
PLWH: people living with HIV
WRAT-4: Wide Range Achievement Test
